# Should medical teachers spend more time modelling or coaching students? A dual eye‐tracking and randomised controlled study on peer instruction in sonography

**DOI:** 10.1111/medu.15725

**Published:** 2025-05-22

**Authors:** Dogus Darici, Hendrik Ohlenburg, Lukas Jürgensen, Cihan Papan, Anita Robitzsch, Markus Missler, Bertrand Schneider

**Affiliations:** ^1^ Institute for Anatomy and Neurobiology University of Münster Münster Germany; ^2^ Institute of Education and Student Affairs, Studienhospital Münster University of Münster Münster Germany; ^3^ General Orthopaedics and Tumour Orthopaedics University Hospital Münster Münster Germany; ^4^ Insistute for Hygiene and Public Health University Hospital Bonn Bonn Germany; ^5^ Clinic for Psychosomatic Medicine and Psychotherapy LVR‐University Hospital Essen Essen Germany; ^6^ Harvard Graduate School of Education Cambridge Massachusetts USA

## Abstract

**Background:**

When first introducing medical procedures, instructors must decide how much of their limited time must be allocated between modelling (demonstrate and explain) and coaching (scaffold and support) students. Given the time constraints in clinical routine, it is currently unknown which relative proportion of modelling versus coaching is more efficient for procedural learning.

**Methods:**

We randomly assigned 73 students without prior knowledge to either an extended modelling (EM) or an extended coaching (EC) group for an emergency sonography training. In the EM group, medical teachers demonstrated a routine examination explaining their thought process, while also providing some coaching. In the EC group, students trained more independently with consistent teacher support, with less emphasis on modelling. We used dual mobile eye‐tracking and voice recording to objectify the teacher–student interactions and applied a comprehensive assessment to understand *which* learning domains improved under which condition.

**Results:**

On post‐tests, the EC group outperformed the EM group by 12% in interpreting dynamic sonographic imagery (*p* = 0.014). They completed the ultrasound examinations 7% faster (*p* = 0.050). There was no statistical difference between the two groups in interpreting static sonographic imagery (*p* = 0.322) nor in practical scores (*p* = 0.062). Contrary to expectations, there were no differences between the groups in terms of eye movement metrics that explained the performance effects. However, two behavioural variables were positively related with learning outcomes across both groups: the percentage of joint visual attention between teacher and student (*β* = 0.316, *p* < 0.001) and the number of words spoken during the training (*β* = 0.175, *p* = 0.004).

**Conclusion:**

This study provides empirical evidence that EC may be particularly effective when introducing new procedural medical skills. In learning complex procedures, direct sensorimotor experience with guided support appears more advantageous than extended observation. These findings suggest that medical educators should give students more opportunities for supervised hands‐on practice rather than relying primarily on demonstration‐based teaching.

## Abstract 2

When first introducing new procedural skills, direct sensorimotor experience with guided support appears more beneficial than extended observation.

## 1 INTRODUCTION

The complexity of medical education demands effective and efficient teaching methods to prepare specialists who can meet tomorrow's healthcare challenges. At the core of medical pedagogy lies a fundamental challenge known as the transfer debate: Students often struggle to apply knowledge gained in classroom or lecture settings to real‐world clinical situations.^1^, ^2^ While classroom‐based education offers efficiency—allowing one teacher to instruct many students simultaneously—it frequently falls short in preparing students for the complexities of clinical practice.^3^ Traditional apprenticeship models address this transfer problem through contextualised learning in authentic settings,^4^ but at a significant cost: They typically require one‐on‐one instruction, making them resource intensive and difficult to scale.^5^

This tension between educational effectiveness and resource efficiency has led to increased attention to how instructional time is utilised within medical apprenticeships.^6^, ^7^ Within apprenticeships, teachers must make critical decisions about how to allocate their limited contact hours with students. In clinical reality, two fundamental teaching approaches have emerged as dominant strategies: modelling (where teachers demonstrate and explain procedures) and coaching (where teachers provide scaffolding and support while students practise independently).^3^ Despite their widespread adoption,^8^, ^9^, ^10^, ^11^ empirical evidence comparing the effectiveness of these approaches remains surprisingly limited, particularly in the context of teaching important medical skills like sonography. To address this gap, our study experimentally evaluates how extended modelling (EM) and extended coaching (EC) approaches affect learning outcomes in procedural training.

### 1.1 Theoretical introduction

Cognitive apprenticeship (CA) is rooted in theories of situated learning, viewing knowledge as dynamically constructed within social contexts and emphasising learning as a social activity profoundly shaped by the setting. The theory of CA was introduced by Collins et al.^12^ to address the above‐mentioned disconnect between school‐taught knowledge and real‐world application, advocating for teaching methods that integrate both cognitive and practical skills needed for expertise.

The theoretical foundation for the current investigation lies in CA theory, which emerged as a response to the limitations of traditional classroom instruction.^12^ CA has gained significant attention in medical education over the past few decades, implemented through various formats including structured clinical rotations,^8^ case‐based learning sessions with expert think‐alouds,^13^ simulation scenarios with detailed debriefings^14^ and peer teaching opportunities.^15^

The CA framework is comprehensive, encompassing four dimensions: content (factual, conceptual and tacit knowledge), method (teaching strategies), sequence (increasing complexity and diversity of tasks) and sociology (situated learning, community of practice and intrinsic motivation). Within the method dimension, Collins et al.^12^ outlined six essential teaching approaches: modelling, coaching, scaffolding, articulation, reflection and exploration. These methods are organised along a continuum that gradually shifts responsibility from teacher to learner, supporting the development of expertise through different mechanisms.

In medical education contexts, all dimensions of CA are likely to play a role in developing clinical expertise. For the purposes of our current investigation, we focus specifically on two teaching methods within the broader CA framework: modelling and coaching. Modelling allows experts to externalise their cognitive processes, demonstrating not just what to do but also how to think through clinical procedures. Through verbal explanations accompanying demonstrations, teachers can highlight crucial aspects of their decision‐making process, helping students understand the rationale behind specific actions. Coaching, conversely, enables students to actively engage with the material while receiving feedback and guidance, potentially fostering deeper learning through hands‐on experience and guided reflection.

While scaffolding, articulation, reflection and exploration are also integral dimensions of the method dimension, we concentrate on modelling and coaching for three reasons: First, path analyses have shown these dimensions to be most proximal in the learning cascade, directly influencing the more distal dimensions articulation and exploration.^16^ Second, modelling and coaching mostly depend on teacher initiative, whereas the other dimensions reflect more self‐regulated activities of the students.^16^ Third, structural equation modelling does not support an independent dimension ‘scaffolding’,^16^ potentially due to its conceptual overlap with other dimensions, particularly modelling and coaching. This makes the modelling–coaching trade‐off particularly critical for instructors' time allocation decisions.

### 1.2 Dual eye‐tracking methodology and voice recording

Despite the recognised importance of modelling and coaching, they have traditionally been assessed through self‐report measures and retrospective surveys.^16^ However, subjective assessments are prone to recall bias, social desirability effects and limited temporal resolution (i.e., the ability to capture fine‐grained, moment‐to‐moment changes in behaviour or interactions over time). Furthermore, participants often struggle to accurately evaluate their behaviours retrospectively, particularly given that many crucial (teaching and learning) behaviours occur implicitly and may not be fully accessible for later reporting. To address these methodological challenges, we implemented a sensor‐based approach, including dual eye tracking and voice recording, to objectively capture both observational patterns during apprenticeship and verbal interactions during CA.

Dual eye tracking, which simultaneously records the visual attention of both teacher and student, offers insights into *how* knowledge is transferred during clinical training.^17^ ^,^ ^18^ This technology allows researchers to quantify important aspects of teacher–student interaction, such as joint visual attention (JVA)—defined as the temporal and spatial coordination of visual attention between teacher and student towards the same elements in their shared visual environment. JVA has been associated with positive learning outcomes^18^, ^19^ but has remained largely unexplored in medical education research.

With the triangulation of visual attention data, voice recording provides crucial information about the verbal dimension of CA.^9^ The combination of eye tracking and voice recording creates a comprehensive picture of the teaching–learning interaction, capturing both the visual and verbal aspects of CA.^19^ This multimodal approach allows us to analyse how teachers verbalise their thinking processes during modelling, how they provide feedback during coaching and how these verbalisations interact with patterns of visual attention. Such detailed analysis could reveal subtle yet important differences that were previously impossible to detect between modelling and coaching approaches, particularly in understanding how verbal guidance influences where and what students attend to during learning.

### 1.3 The current study

While research has established CA's overall effectiveness in bridging the theory–practice gap,^3^ less is known about the relative impact of its different components. Given that we cannot realistically implement full CA approaches for teaching every aspect of medicine, we need to understand which elements are most effective when introducing students to new concepts and skills, when is it more valuable to have experts demonstrate their thinking processes and when should they step back and guide student practice instead.

Our study addresses this gap by comparing the effectiveness of modelling versus coaching in medical education, using dual eye‐tracking methodology and voice recording to provide empirical evidence for optimising CA implementation. We hypothesise that modelling and coaching will yield distinct patterns in visual attention between teachers and students and learning outcomes.

#### 1.3.1 Hypothesis 1

In the EM condition, we anticipate a high degree of JVA between the teacher and student, particularly on critical areas of interest (AOIs) such as the ultrasound monitor. As teachers demonstrate and explain each action, we hypothesise that students are likely to mirror their gaze, following the teachers' focal points. This mirroring is expected to help students understand the procedural flow and the rationale behind specific clinical decisions.^20^ Consequently, we expect that students in the EM group will exhibit enhanced accuracy in sonographic image interpretation when assessed on related tasks.

#### 1.3.2 Hypothesis 2

In the EC condition, where students are encouraged to perform tasks independently while receiving guidance, we expect a more varied visual attention pattern and a lower JVA. Students in this group may focus more on their own hands, equipment or areas they find challenging. Teachers may direct their gaze between the students' actions and critical AOIs to provide corrective feedback. This ‘less synchronized gaze pattern’ is hypothesised to foster self‐guided exploration and problem‐solving, allowing students to build autonomy in handling the procedure while benefiting from targeted feedback.^21^ We anticipate that this increased autonomy will enhance students' practical performance and skill retention.

Additionally, we examine how these teaching approaches differentially affect students' cognitive load, as modelling and coaching may impose distinct demands on learners' cognitive resources. For example, modelling may reduce extraneous cognitive load (ECL) through clear demonstration but potentially limit germane (i.e., beneficial) processing through passive observation, while coaching might initially increase cognitive load but enhance learning through active engagement. By measuring cognitive load alongside performance and eye‐tracking data, we can better understand the cognitive mechanisms underlying the effectiveness of different teaching approaches in medical education.

This research aims to help medical educators make evidence‐based decisions about allocating their limited time and resources, ultimately leading to more efficient and effective medical training that successfully bridges the gap between theoretical knowledge and practical application.

## 2 METHODS

This two‐group randomised controlled study was conducted at the medical faculty of the University of Münster, Germany, with data collection from April 2023 to February 2024. This research project received approval from the ethics board (2023‐439‐f‐N) and was conducted in accordance with the Declaration of Helsinki and its later amendments. Informed consent was obtained from all participants.

### 2.1 Participants

Participants as learners were recruited through email postings and in‐course announcements at the University of Münster, with self‐selected registration through an online link. Based on prior dual eye‐tracking research on JVA,^17^ we conducted an a priori power analysis with an estimated effect size of *d* = 0.8 and a power of 0.80, targeting 26 dyads per group. Our teaching team consisted of four medical students from higher semesters (7–10; each with at least 1 year of ultrasound teaching experience) and one resident doctor, all recruited through word of mouth.

Study participation did not affect course grades, and no monetary incentives were provided. The opportunity to gain hands‐on ultrasound experience served as a natural incentive. To increase external validity, we welcomed novices from all health profession programmes at the faculty, including medical, midwifery, cognitive neuroscience and physician assistant students. We excluded students who had previously completed a full sonography course, typically those above their seventh semester of medical studies. Although we encouraged students wearing glasses to use contact lenses during the trials to optimise eye‐tracking accuracy, neither glasses nor red–green blindness served as an exclusion criterion.

### 2.2 Study procedure

The study focused on practical emergency sonography training, specifically FAST (Focused Assessment with Sonography for Trauma). This procedure focuses on identifying free fluid, often a sign of haemorrhage or organ damage, in four key anatomical regions. We selected this technique due to its relevance in clinical medicine and complexity, which typically induces high intrinsic cognitive load (ICL) for novice learners.^22^ The challenges include probe manoeuvring, hand–eye coordination, dynamic image interpretation and the need to comprehend abstract, four‐dimensional sonographic images from multiple angles.

The complete protocol spanned approximately 75 min and took place in the university's simulation and skills training centre (‘Studienhospital’). Each parkour station lasted 12 min, with an additional 3 min allocated for rotation, ensuring equal time on task across participants. We incorporated regular rest phases after four run‐throughs to prevent teacher fatigue. We ensured that each of the teachers taught both conditions equally often. Participants were randomised in the two groups via *simple random allocation* using the Rv.uniform command in SPSS (Version 29, IBM Corp., Armonk, NY, USA). The learners were blinded to their respective conditions. Teachers underwent a 30‐min training session to harmonise learning objectives and familiarise themselves with time restrictions, studying testing materials beforehand to ensure constructive alignment of their teaching. The participants carried a preconfigured tablet throughout the study (i.e., a university internal survey programme to store data and to give standardised instructions to the participants, see Table S1).

Participants progressed through six stations in different rooms: First, they completed sociodemographic questionnaires. Second, they studied a theoretical FAST sonography text, including standard sonographic and schematic images for probe positioning, and the rationale behind the examination. In the third station, participants were randomly assigned to either modelling‐based or coaching‐based training (see Section 2.3), during which we employed the dual mobile eye‐tracking methodology and voice recording. The fourth station involved completing cognitive load questionnaires^23^ and teaching quality assessments (Medical Clinical Teaching Questionnaire [MCTQ]).^16^ In the fifth station, participants underwent an objective structured clinical examination (OSCE) administered by an independent ‘testing teacher’ who was blinded to participants' group assignment, ensuring unbiased assessment. Finally, we assessed participants' anatomy knowledge and static image interpretation abilities (see Section 2.4).

### 2.3 Modelling‐ and coaching‐based trainings

Our study design acknowledged that both modelling and coaching are essential components of CA, making it impractical and potentially detrimental to eliminate either element. Instead, we adopted a comparative approach that varied the relative emphasis of each component while maintaining the integrity of the training framework. Therefore, the trainings were structured as follows:

- EM group: 9‐min modelling + 3‐min coaching
- EC group: 3‐min modelling + 9‐min coaching

**FIGURE 1:**
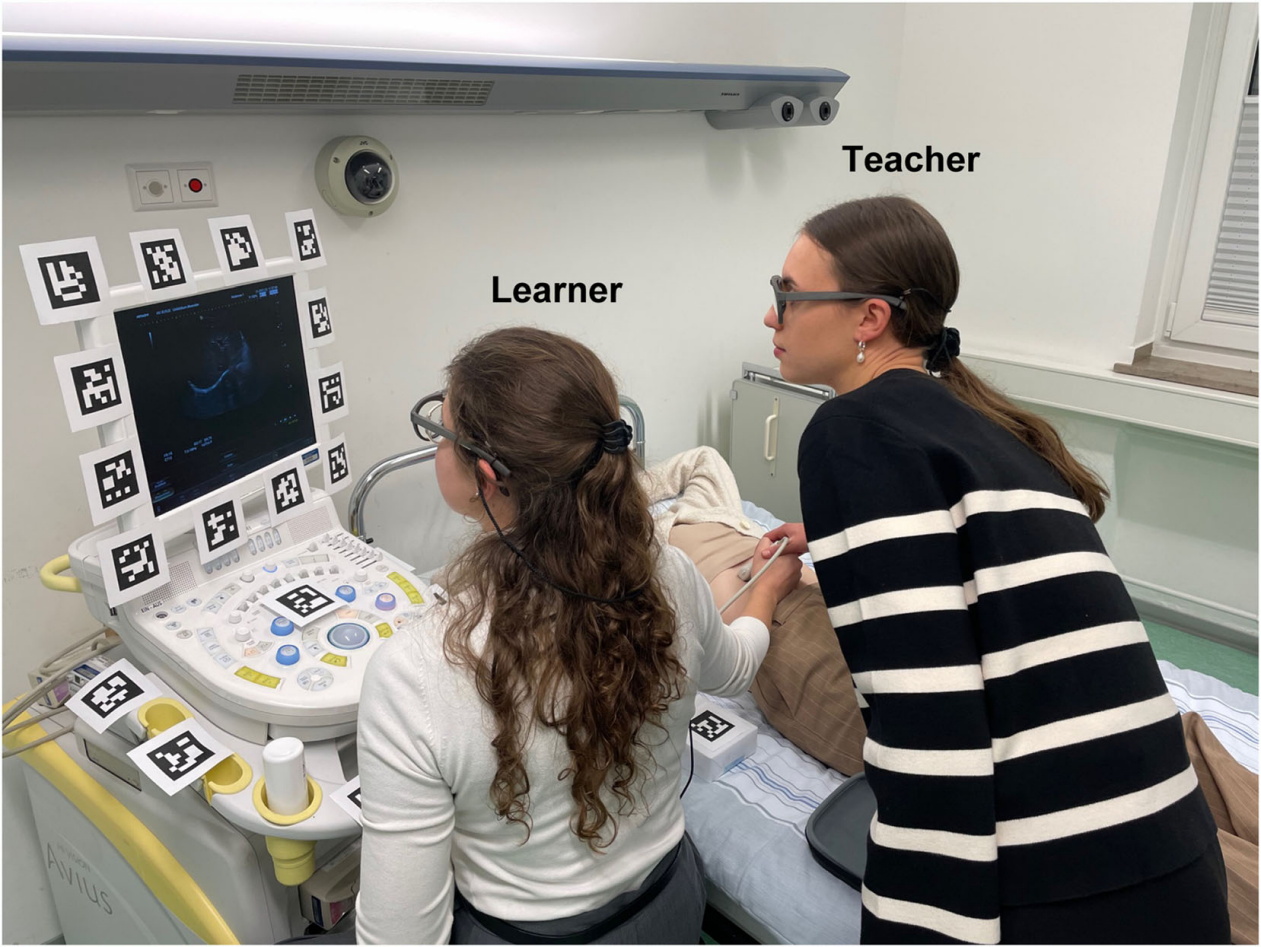
Training setup and post‐test assessments. The picture shows the training setup in a coaching scenario, where the learner is performing the ultrasound examination independently while receiving scaffolding and support from the teacher. Note that both the teacher and learner wore mobile eye‐tracking glasses to track their eye movements during the training. [Color figure can be viewed at wileyonlinelibrary.com]

The 12‐min total training duration, while brief for mastering ultrasound examination skills,^24^ was deliberately chosen to prevent ceiling effects and highlight differences between training formats. Sessions involved three participants: one teacher, one learner and one simulation patient (Figure 1). We opted for simulation patients rather than mannequins or static models because FAST sonography is a dynamic assessment that requires adapting to respiratory movements and patient compliance, which can be best simulated with a live person. Researchers observed through a mirrored glass to address technical issues. Training components included the following:

- Modelling: The teacher performs the examination and explanation while the learner observes.
- Coaching: The learner performs the examination while receiving the teacher's guidance and correction.

### 2.4 Data sources

We collected multiple types of data to comprehensively assess the effectiveness of both training approaches. Performance data (primary outcomes) included practical skills assessment through an OSCE^25^ comprising four subsections, with performance rated according to a standardised checklist (averaged four items, Cronbach's *α* = 0.71). We evaluated both dynamic image interpretation skills (averaged four items, *α* = 0.69) and static image interpretation skills (averaged 16 items, *α* = 0.71), with the former assessed during the OSCE and the latter through standardised sonographic images. Task completion time (later transformed into time efficiency, with the fastest time being equal to 100%) was measured during the OSCE using a stopwatch. Additionally, we assessed participants' prior knowledge in anatomy through single‐choice questions (averaged 15 items, *α* = 0.71).

For physiological data collection, we employed dual mobile eye tracking using two Pupil Labs Neon glasses operating at 200 Hz, with precision and accuracy values of 1.3° after offset correction,^26^ including a scene camera recording at 1600 × 1200 (30 Hz). These devices allowed us to capture detailed gaze behaviour during the training sessions. We analysed the most basic eye movement parameters, including fixation count, with particular attention to fixation on defined AOIs. Additionally, we focused on JVA, calculated with cross‐recurrence quantification analysis as described by Coco and Dale,^27^ to understand synchronised viewing patterns between teachers and learners. To optimise light conditions and ensure reliable eye‐tracking data, all training sessions were conducted in a dedicated laboratory space with controlled ambient lighting (350 lx; LX1010BS). Data quality for each recording was manually reviewed.

To capture the verbal dimension of the training, we recorded all audio during the sessions via microphones incorporated into the eye‐tracking glasses (16‐bit resolution). The number of words spoken during the training was then extracted using the tuneR package in R.^28^ These recordings provided data about the quantity of verbal interactions between tutors and learners.

Questionnaire data included basic demographic information (age, gender and current semester of study) and self‐assessments. The Cognitive Load Questionnaire^23^ helped us assess participants' mental effort and cognitive load during training. This instrument consisted of five items including two factors (ICL and ECL), including questions such as ‘This task was very complex’, and demonstrated moderate to good internal consistencies (*α* = 0.79 for ICL, *α* = 0.62 for ECL). Teaching quality was evaluated using the MCTQ,^16^ which we translated into German from its original version. The MCTQ subscales modelling (*α* = 0.48) and coaching (*α* = 0.59) comprised three items, respectively, including questions such as ‘The clinical teacher consistently demonstrated how to perform the clinical skill’.

### 2.5 Analyses

For our statistical analyses, we used RStudio V. 2023.12.1 (Posit Software, Boston, MA, USA). Initial data preprocessing included outlier analysis and participant exclusion (*n* = 2 dyads) based on predetermined criteria using the Grubbs test. We conducted univariate analyses of variance (anovas) to examine group differences, while a generalised linear mixed model (GLMM) allowed us to account for the nested structure of our data. For this, we included the teachers as random factors and group allocation as fixed effects. The variables gender, age, semester, training disciplines, prior knowledge in anatomy and prior knowledge in sonography were included as covariates. We employed multiple regression analyses to investigate predictive relationships. For better comparison, we normalised the performance data (0%–100%).

All statistical tests were conducted with a significance level of *α* = 0.05, and we report appropriate effect sizes and confidence intervals for significant results. Before conducting our primary analyses, we verified all necessary statistical assumptions, including normality of distributions, homogeneity of variances and independence of observations. In addition, we applied a bootstrap analysis (*N* = 5000 samples) in cases where normality distribution was violated. Data visualisation was accomplished using RStudio V. 2023.12.1 (Posit Software, Boston, MA, USA).

## 3 RESULTS

### 3.1 Participant characteristics

We recruited *N* = 73 students from the University of Münster across four different subjects: medicine, physician assistant, midwifery and neuroscience. Participants in this study were randomly allocated into two groups: the EM group (*n* = 37 dyads) and the EC group (*n* = 36 dyads). All participants received the treatments per protocol, and 73 participants were incorporated into the final analysis (Table 1).

**TABLE 1:** Baseline characteristics of participants after randomisation (simple random allocation).

Variable	EM group (*n* = 37 dyads)	EC group (*n* = 36 dyads)	Significantly different?
Age (years)	22.41	21.97	*t*(71) = 0.54, *p* = 0.594
Gender			*χ* ^2^(1) = 0.04, *p* = 0.947
Men	9	9	
Women	28	27	
Diverse	0	0	
Training disciplines			*χ* ^2^(3) = 4.19, *p* = 0.241
Medicine	30	32	
Physician assistant	3	0	
Midwifery	0	1	
Neuroscience	4	3	
Semester of study			*χ* ^2^(4) = 1.61, *p* = 0.808
1	8	4	
2	2	3	
3	13	14	
4	10	11	
5	4	4	
Wearing glasses^a^			*χ* ^2^(1) = 2.10, *p* = 0.147
Yes	6	11	
No	31	25	
Red–green blindness			*χ* ^2^(1) = 2.11, *p* = 0.146
Yes	0	2	
No	37	34	

*Note*: No group differences were found, indicating successful randomisation.

Abbreviations: EC, extended coaching; EM, extended modelling.

^a^ Does not imply contact lenses.

Sociodemographics were analysed to confirm baseline similarities, with comparisons conducted on age, gender, training disciplines, semester of study and the presence of specific items, such as wearing glasses and red–green colour blindness. In summary, the characteristics analysed for participants in both the EM and EC groups were statistically comparable across all measured demographic and background variables. This balance supports the validity of subsequent group comparisons in study outcomes.

### 3.2 Effects of modelling and coaching on learning outcomes

**FIGURE 2:**
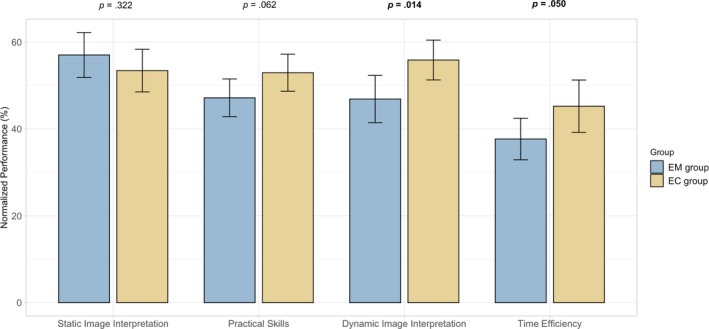
Effects of modelling and coaching on learning outcomes. Bar charts showing the mean and standard deviation and the results of the mean comparison as *p*‐values. The groups that received the modelling and coaching trainings are shown in sky blue and copper, respectively. Static image interpretation refers to the ability of students to interpret sonoanatomical structures on non‐moving images. Practical skills refer to the accuracy of conducting a sonographic examination as measured with a checklist (*objective structured clinical examination*). Dynamic image interpretation refers to the ability of students to find and interpret sonoanatomical structures on the ultrasound monitor during the dynamic practical task. Time efficiency refers to the speed of the practical examination. *Note*: Performance measures were normalised (0%–100%) to allow a better comparison. [Color figure can be viewed at wileyonlinelibrary.com]

Using a GLMM, we investigated the effects of modelling and coaching on various learning outcomes in sonography (Figure 2).

#### 3.2.1 Static image interpretation

Students in the EM group (*M* = 57.0% ± 26.1) and the EC group (*M* = 53.4% ± 23.1) displayed similar static image interpretation performances, with no significant difference found, *F*(1, 186) = 0.984, *p* = 0.322.

#### 3.2.2 Practical skills

Students in the EC group (*M* = 52.9% ± 20.2) outperformed the EM group (*M* = 47.1% ± 21.9); however, the main effect did not reach significance, *F*(1, 186) = 3.52, *p* = 0.062.

#### 3.2.3 Dynamic image interpretation

The EC group (*M* = 55.8% ± 21.6) outperformed the EM group (*M* = 46.9% ± 27.4) in dynamic image interpretation, *F*(1, 186) = 6.12, *p* = 0.014, partial *η* ^2^ = 0.031, indicating a small to medium effect size.

#### 3.2.4 Time efficiency

In terms of time efficiency (i.e., how fast participants solved the practical task), the EC group (*M* = 45.2% ± 28.4) performed better than the EM group (*M* = 37.7% ± 24.1), *F*(1, 186) = 3.89, *p* = 0.050, partial *η* ^2^ = 0.021.

### 3.3 Effects of modelling and coaching on visual attention

We analysed dual eye‐tracking data collected during the training to examine differences in visual attention between the two training groups.

#### 3.3.1 Fixations on AOIs (Figure 3a)

**FIGURE 3:**
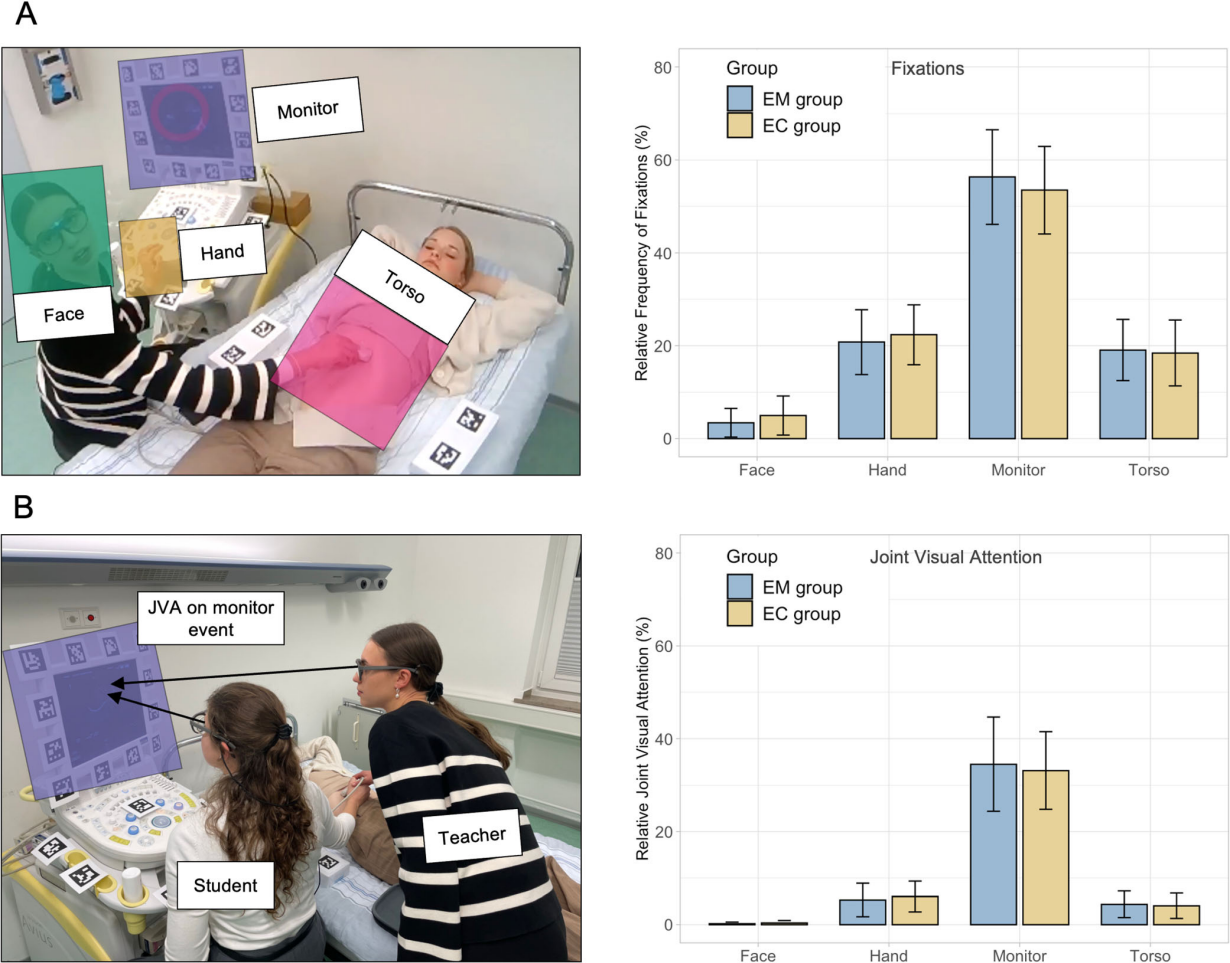
Results of the dual mobile eye‐tracking data during training are shown. (a) Frequencies of fixations (i.e., focus on a target) of the teacher and students on the four critical AOIs (monitor, hand, face and torso) are shown for the EM group (sky blue) and EC group (copper) separately. (b) The data also demonstrate the frequencies of simultaneous area fixation (joint visual attention) between groups. For example, JVA on the face represents events where both participants look at each other's face at the same time. [Color figure can be viewed at wileyonlinelibrary.com]

Overall, no significant group differences were observed in the fixation count for the AOIs: face (teacher/learner), hand, monitor and torso. However, we observed a tendency for the EM group to focus more frequently on the monitor. Across both groups, the participants tended to fixate most often on the ultrasound monitor (55% ± 10.5), followed by fixations on the hand (21% ± 6.7), torso (19.0% ± 6.8) and least frequently on the face (4% ± 3.8). The most frequent transitions between AOIs occurred from the hand to the monitor (Figure S1), indicating cueing events (i.e., finger pointing).

#### 3.3.2 JVA on AOIs (Figure 3b)

We also analysed JVA, representing the percentage of time both teacher and student simultaneously focused on the same AOI. No significant differences were found between both groups. While the distribution of visual attention across AOIs was similar to that observed in individual fixation counts, JVA events occurred less frequently overall. Additionally, the proportion of JVA on specific AOIs shifted, with a notable emphasis on the monitor. JVA events on the face (i.e., instances where the teacher and student looked at each other) were exceedingly rare, accounting for less than 1% of all fixations.

### 3.4 Effects of modelling and coaching on cognitive load and clinical teaching

Next, we applied a GLMM to assess how the training affected students' perceived cognitive load and clinical teaching effectiveness (Table 2). The results revealed one significant difference: Students in the EC group gave lower ratings on the ‘modelling’ subscale of the MCTQ compared to those in the EM group, *F*(1, 43) = 3.87, *p* < 0.001, *η* ^2^ = 0.153. Contrary to expectations, the EC group did not rate the ‘coaching’ or ‘exploration’ subscale more favourably than did the EM group. Besides, the overall values were high for ICL and low for ECL.

**TABLE 2:** Effects of modelling and coaching on cognitive load and clinical teaching.

Variable	EM group (*n* = 37 dyads)	EC group (*n* = 36 dyads)
	*Mean* ± SD	*Mean* ± SD
Intrinsic cognitive load	4.08 ± 1.45	3.95 ± 1.12
Extrinsic cognitive load	1.82 ± 0.68	2.00 ± 0.80
Germane cognitive load	4.12 ± 1.35	4.32 ± 1.23
Clinical teaching (MCTQ)	4.01 ± 0.29	3.96 ± 0.49
Modelling	4.73 ± 0.35*	4.36 ± 0.56*
Coaching	4.59 ± 0.45	4.50 ± 0.60

Abbreviation: MCTQ, Maastricht Clinical Teaching Questionnaire (Likert scale 1–5).

* Significantly different at the *p* < 0.05 level.

### 3.5 Behavioural and demographic predictors of learning success

Across both groups, multiple regression analyses revealed distinct patterns of predictors across three learning outcomes in sonography training (Table 3).

**TABLE 3:** Multiple regression models to predict learning success in sonography training.

Predictor	*B*	*β*	*p*
Dependent: practical performance			
Fixation count (*n*)	−0.001	−0.015	0.838
Joint visual attention (%)	0.028	0.316	<0.001***
Words spoken (*n*)	0.001	0.122	0.090
Age	0.061	0.297	<0.001***
Gender^a^	−0.064	−0.036	0.585
Semester of study	0.067	0.121	0.279
Training disciplines	−0.266	−0.497	<0.001***
Model fit	*R* ^2^_adj_ = 0.248, *p* < 0.001		
Dependent: static image interpretation			
Fixation count (*n*)	−0.001	−0.004	0.956
Joint visual attention (%)	−0.003	−0.126	0.063
Words spoken (*n*)	−0.001	−0.015	0.825
Age	0.008	0.141	0.067
Gender^a^	0.017	0.036	0.572
Semester of study	0.059	0.377	<0.001***
Training disciplines	−0.040	−0.260	0.020*
Model fit	*R* ^2^_adj_ = 323, *p* < 0.001		
Dependent: Dynamic image interpretation			
Fixation count (n)	0.001	−0.068	0.262
Joint visual attention (%)	0.015	0.274	<0.001***
Words spoken (n)	0.001	0.175	0.004**
Age	0.023	0.188	0.005**
Gender^a^	0.237	0.223	<0.001***
Semester of study	0.023	0.067	0.471
Training disciplines	−0.231	−0.709	<0.001***
Model fit	*R* ^2^_adj_ = 0.475, *p* < 0.001		

Abbreviations: *B*, unstandardised regression coefficient; *β*, standardised regression coefficient; *R* ^2^_adj_, coefficient of determination adjusted for multiple predictors.

^a^ Positive values favour men.

*** *p* < 0.001.

** *p* < 0.01.

* *p* < 0.05.

For practical performance (*R* ^2^_adj_ = 0.248, *p* < 0.001), JVA (*β* = 0.316, *p* < 0.001), age (*β* = 0.297, *p* < 0.001) and training disciplines (*β* = −0.497, *p* < 0.001) emerged as significant predictors. Static image interpretation (*R* ^2^_adj_ = 0.323, *p* < 0.001) was primarily predicted by the semester of study (*β* = 0.377, *p* < 0.001) and training disciplines (*β* = −0.260, *p* = 0.020). Dynamic image interpretation showed the strongest model fit (*R* ^2^_adj_ = 0.475, *p* < 0.001), with significant contributions from JVA (*β* = 0.274, *p* < 0.001), words spoken (*β* = 0.175, *p* = 0.004), age (*β* = 0.188, *p* = 0.005), gender (*β* = 0.223, *p* < 0.001) and training disciplines (*β* = −0.709, *p* < 0.001). Notably, the fixation count did not predict any learning outcome.

## 4 DISCUSSION

Our study provides insights into the relative effectiveness of modelling and coaching approaches in medical education, particularly when first introducing procedural skills like sonography. The results challenge some of our initial hypotheses while offering implications for medical education practice and theory. We discuss our main findings below.

### 4.1 Balancing modelling and coaching in procedural tasks

Our data revealed that the EC group performed slightly better than the EM group on certain dimensions, especially on tasks that required dynamic image interpretation and time efficiency. While these effect sizes were small to medium, they should be considered in the context of our study design, which compared two active educational interventions rather than an intervention versus no treatment. In such comparative effectiveness studies, even small to medium effects can be meaningful and educationally relevant.^29^ However, we note the absence of significant differences in static image interpretation and practical scores, suggesting that the advantages of EC may be task specific.

These results indicate that for some procedural skills, active engagement may offer modest advantages over observational learning. We think that while the EM group could observe the correct procedures, the EC group gained additional firsthand sensorimotor experience during the training, which may have contributed to the performance differences on dynamic tasks.

A particularly intriguing finding was the EC group's time efficiency despite similar visual attention patterns to the EM group. This suggests that the benefits of coaching may lie not in directing visual attention differently, but in more effectively translating visual information into motor actions. Drawing on theories of perception–action coupling,^30^ we speculate that the coaching approach might facilitate more efficient integration of perceptual input with motor responses.

Third, the absence of significant group differences in static image interpretation provides an important context for understanding *when* different teaching approaches might be most effective. When tasks involved only visual analysis without motor components, the EC group's advantage vanished or reversed, indicating that their superior performance in dynamic tasks might be due to enhanced embodied understanding,^31^ rather than visual processing.

These findings suggest that both modelling and coaching have distinct educational values, with their effectiveness depending on context. Rather than viewing these as competing alternatives, educators should consider how to balance both approaches based on task and specific learning objectives.

### 4.2 The JVA between teacher–learner dyads is associated with procedural learning

We also predicted higher JVA in the EM group, particularly on critical areas like the ultrasound monitor. This hypothesis was partially supported, though in an unexpected way. While both conditions exhibited high JVA, the distinguishing factor was not the teaching approach per se but rather the strength of JVA's association with positive learning outcomes *across* both groups. The strong correlation between JVA and learning outcomes, regardless of teaching condition, indicates that visual synchronisation between a teacher and student may be an important mechanism of knowledge transfer in procedural skill acquisition. This idea is supported by Schneider^19^ and a more recent work in medical simulation training.^20^ While our current study provides preliminary evidence supporting this assumption, the complex interplay between visual attention and procedural learning warrants comprehensive investigation. The potential to reframe educational strategies based on visual synchronisation principles represents a promising avenue for future research.

### 4.3 Implications for medical theory and practice

The study findings have significant implications for both theoretical understanding and practical application in medical education. From a theoretical perspective, the findings suggest a bridge between CA theory and embodied cognition in medical education. While CA emphasises making expert thinking visible through modelling and guided practice, embodied cognition stresses how knowledge is fundamentally grounded in physical experience. The superior performance of the EC group in dynamic tasks suggests that these frameworks may be complementary rather than separate—effective learning appears to happen when expert guidance is integrated with direct sensorimotor experience. This supports an embodied cognition framework for understanding procedural skill acquisition, suggesting that the integration of perceptual and motor learning in sonography may be more crucial than previously recognised. This challenges traditional assumptions about the primacy of observational learning (‘doctor shadowing’) in medical education and may be integrated into newer models of curriculum development, strengthening the rising role of ‘hands‐on’‐based medical education and entrustable professional activities.^32^

Practically, medical educators should prioritise guided hands‐on practice over lengthy demonstrations, particularly when teaching new procedural skills. This shift would allow students to develop crucial sensorimotor experience early in their training, rather than spending extensive time observing before attempting procedures themselves. Additionally, our eye‐tracking findings suggest that JVA could serve as a valuable metric for evaluating teaching effectiveness, offering a new quantitative way to assess the quality of teacher–student interactions during practical training. Also, educators should carefully consider the nature of the skill being taught when selecting their teaching approach. While coaching seems particularly effective for dynamic tasks requiring real‐time coordination, the benefits were less pronounced for static tasks like image interpretation. This suggests that teaching strategies should be tailored to match the specific demands of the skill being taught, rather than applying a *one‐size‐fits‐all* approach to procedural learning. For assessment purposes, this means developing more sophisticated frameworks that recognise the multifaceted nature of procedural learning.

### 4.4 Limitations and future directions

Several limitations should be considered. The relatively brief training duration (12 min) may not fully reflect the learning dynamics of longer term medical training. Additionally, the observed effects may be context dependent and reflect only effects in ultrasound teaching. Thus, further studies are warranted to generalise the findings to other types of medical procedures. Furthermore, since the same tutors taught both conditions, there is potential for adaptive teaching strategies that might have masked the differences between both groups. Finally, eye movement analysis revealed low variance between the AOIs, with participants predominantly focusing on the ultrasound monitor.

It is also important to recognise that the quality of implementation for both modelling and coaching approaches can vary between instructors, assessors and contexts, potentially affecting outcomes. Future research could expand beyond our focused examination of modelling and coaching to investigate how instructors might effectively facilitate the other dimensions of the CA model. Future research should also examine how the expertise gap between the teacher and learner influences the effectiveness of different teaching methods, as our study utilised advanced peer learners rather than established experts in sonography, which may not fully reflect the CA dynamics assumed in traditional expert–novice interactions.

### 4.5 Conclusion

This study suggests that coaching‐based approaches outperform modelling‐based approaches when teaching new procedural skills like sonography. These findings indicate that medical educators should prioritise guided hands‐on practice over extended demonstrations when first introducing new procedural skills.

## AUTHOR CONTRIBUTIONS

**Dogus Darici:** Conceptualization; investigation; funding acquisition; writing—original draft; methodology; visualization; writing—review and editing; formal analysis; project administration; data curation; software; resources; supervision; validation. **Hendrik Ohlenburg:** Project administration; resources; writing—original draft. **Lukas Jürgensen:** Data curation; writing—review and editing; writing—original draft. **Cihan Papan:** Writing—review and editing; methodology; investigation; writing—original draft. **Anita Robitzsch:** Investigation; writing—review and editing; writing—original draft; methodology. **Markus Missler:** Conceptualization; funding acquisition; validation; supervision; resources. **Bertrand Schneider:** Conceptualization; writing—original draft; methodology; validation; writing—review and editing; supervision; resources.

## CONFLICT OF INTEREST STATEMENT

We have no conflicts of interest to disclose.

## ETHICS STATEMENT

This research project received approval from the ethics board (2023‐439‐f‐N) and was conducted in accordance with the Declaration of Helsinki and its later amendments. Informed consent was obtained from all participants.

## Supporting information


**Figure S1.** (A) Panel showing eye movements of one dyad from each training Modelling (sky blue) and Coaching (copper). (B) Panel showing the transitions from one area of interest to another separately for both groups.

**Figure S2.** Station 2 with a theoretical introduction on FAST sonography.

**Figure S3.** Station 6 is shown, where learners interpreted sonographic images to assess their static image interpretation performance.

**Figure S4.** Station 5 is shown, where the learner (left) performed an emergency sonography examination. The tutor (right), who was blinded to the intervention, assessed their practical performance and dynamic image interpretation performance based on a standardised checklist (OSCE).

**Figure S5.** Items for assessing static image interpretation. Learners had to label the correct sonoanatomical names for the numbers.

**Table S1.** Instructions for participants.

## Data Availability

The data that support the findings of this study are available from the corresponding author upon reasonable request.
