# Supplementary figures and images for: Should medical teachers spend more time modelling or coaching students? A dual eye‐tracking and randomised controlled study on peer instruction in sonography
Source: Med Educ. 2025 May 22;59(10):1105–16. doi: 10.1111/medu.15725 (PMC12438010; doi:10.1111/medu.15725)

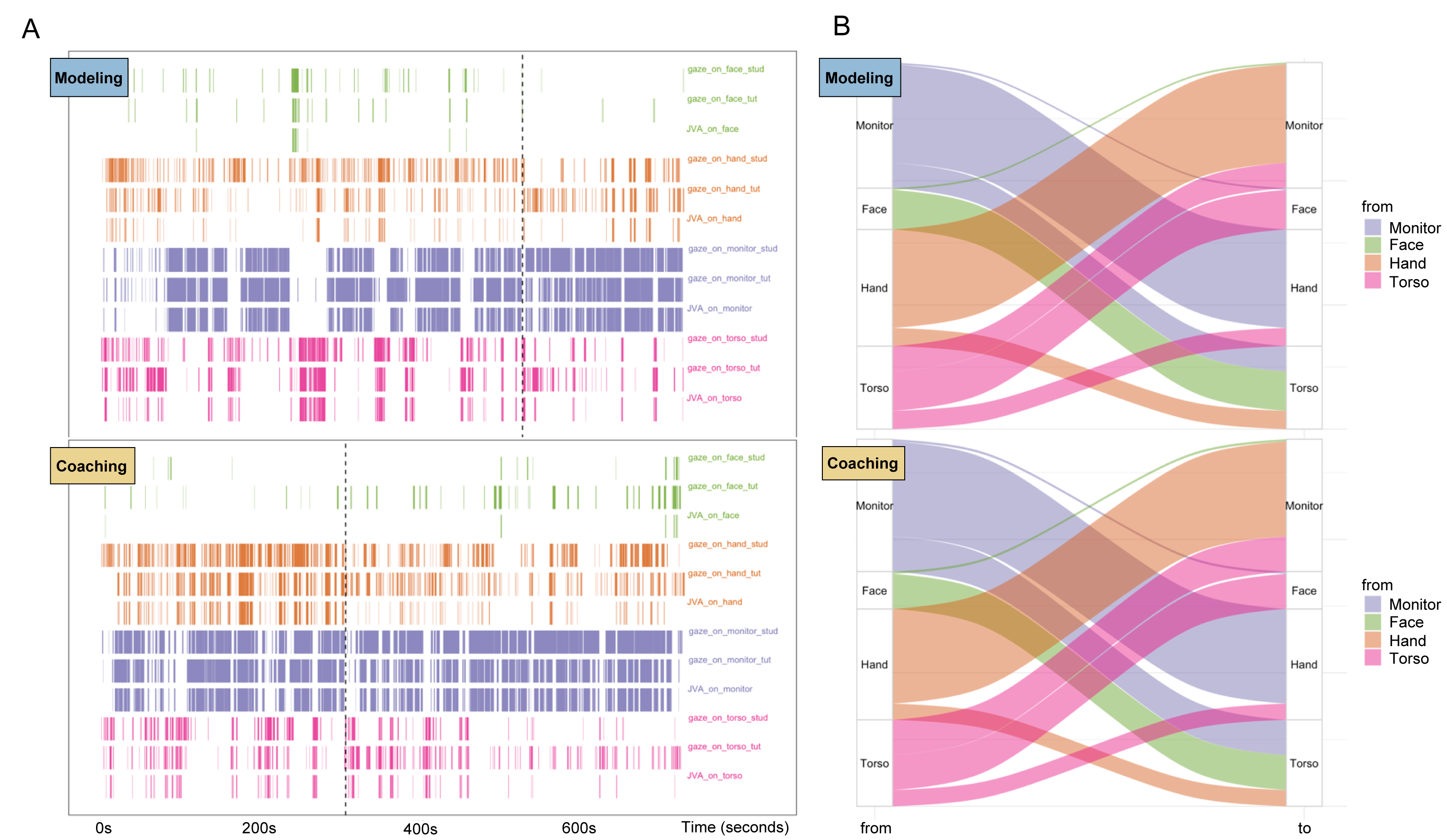

Supplement: Supplementary file 1 — Figure S1. (A) Panel showing eye movements of one dyad from each training Modelling (sky blue) and Coaching (copper). (B) Panel showing the transitions from one area of interest to another separately for both groups. [file MEDU-59-1105-s001.tif]

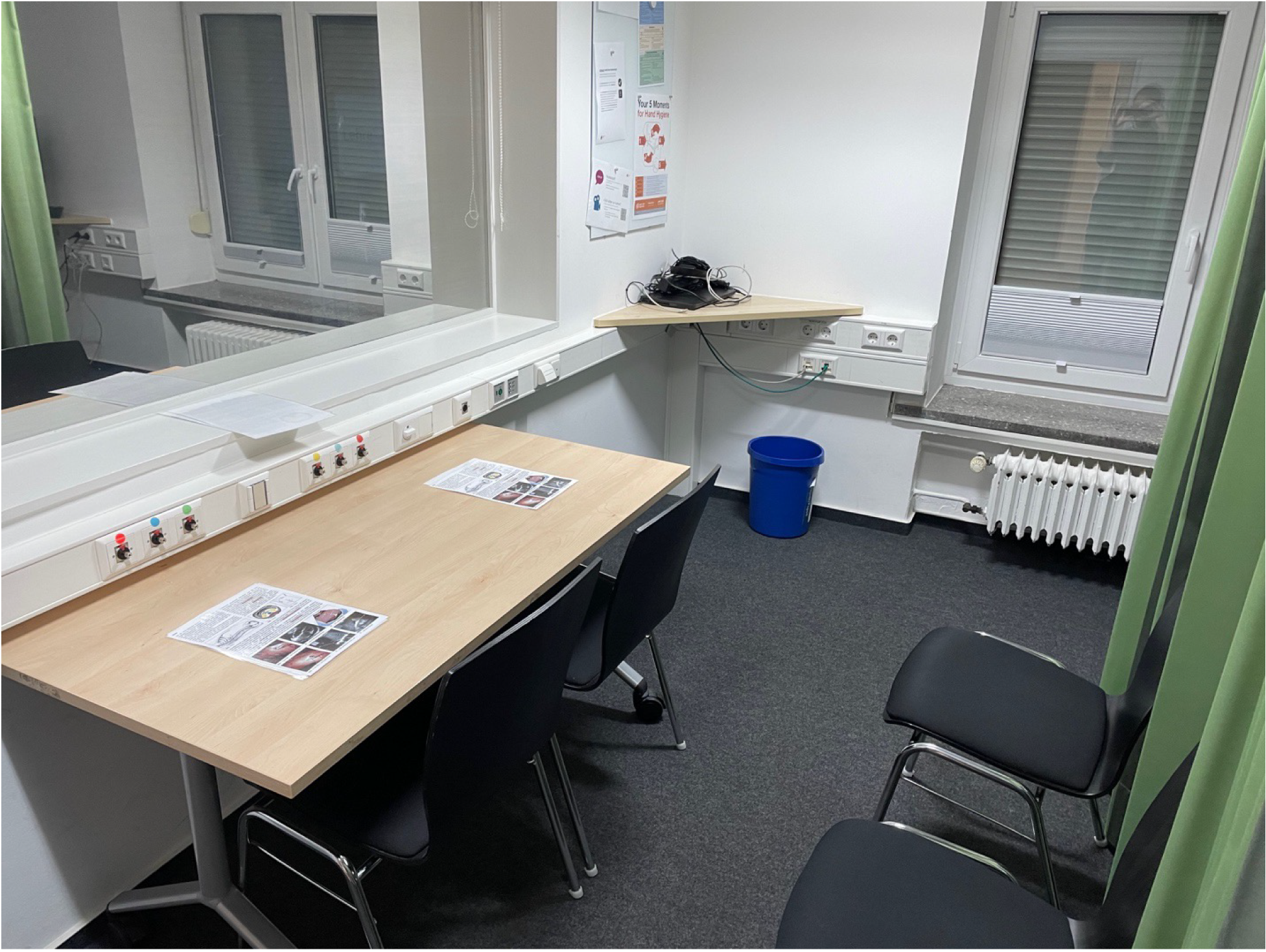

Supplement: Supplementary file 2 — Figure S2. Station 2 with a theoretical introduction on FAST sonography. [file MEDU-59-1105-s002.tif]

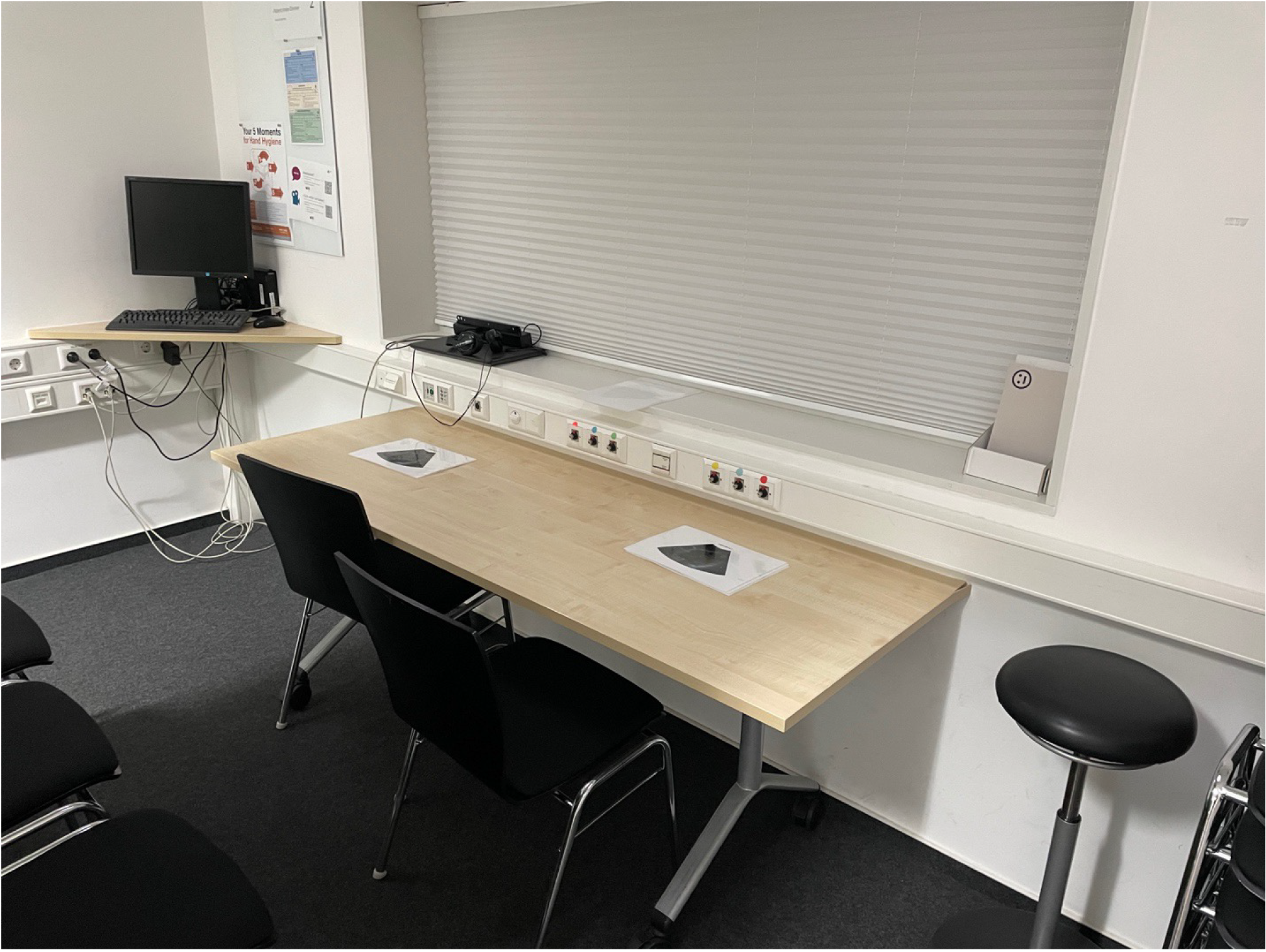

Supplement: Supplementary file 3 — Figure S3. Station 6 is shown, where learners interpreted sonographic images to assess their static image interpretation performance. [file MEDU-59-1105-s004.tif]

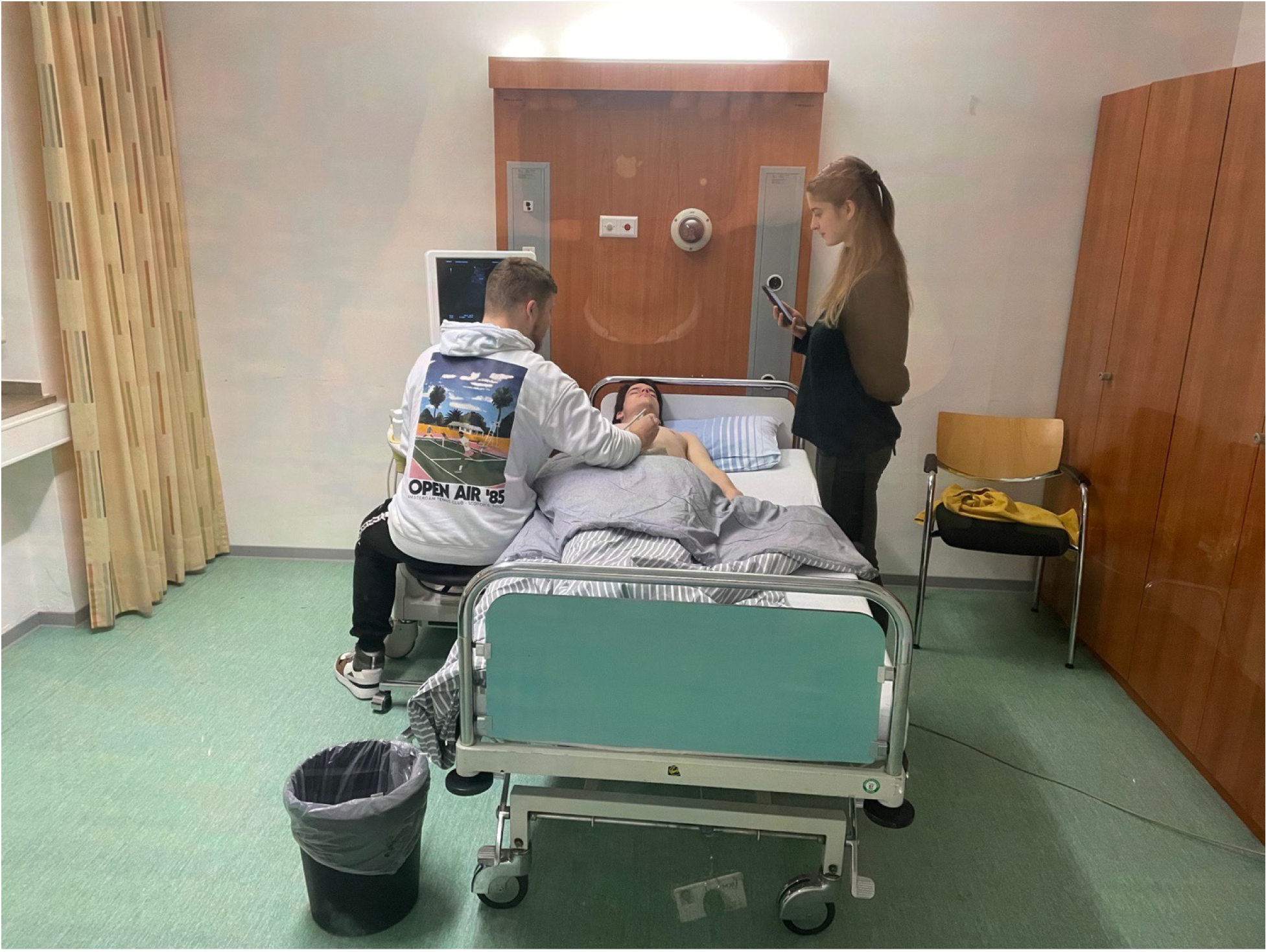

Supplement: Supplementary file 4 — Figure S4. Station 5 is shown, where the learner (left) performed an emergency sonography examination. The tutor (right), who was blinded to the intervention, assessed their practical performance and dynamic image interpretation performance based on a standardised checklist (OSCE). [file MEDU-59-1105-s003.tif]

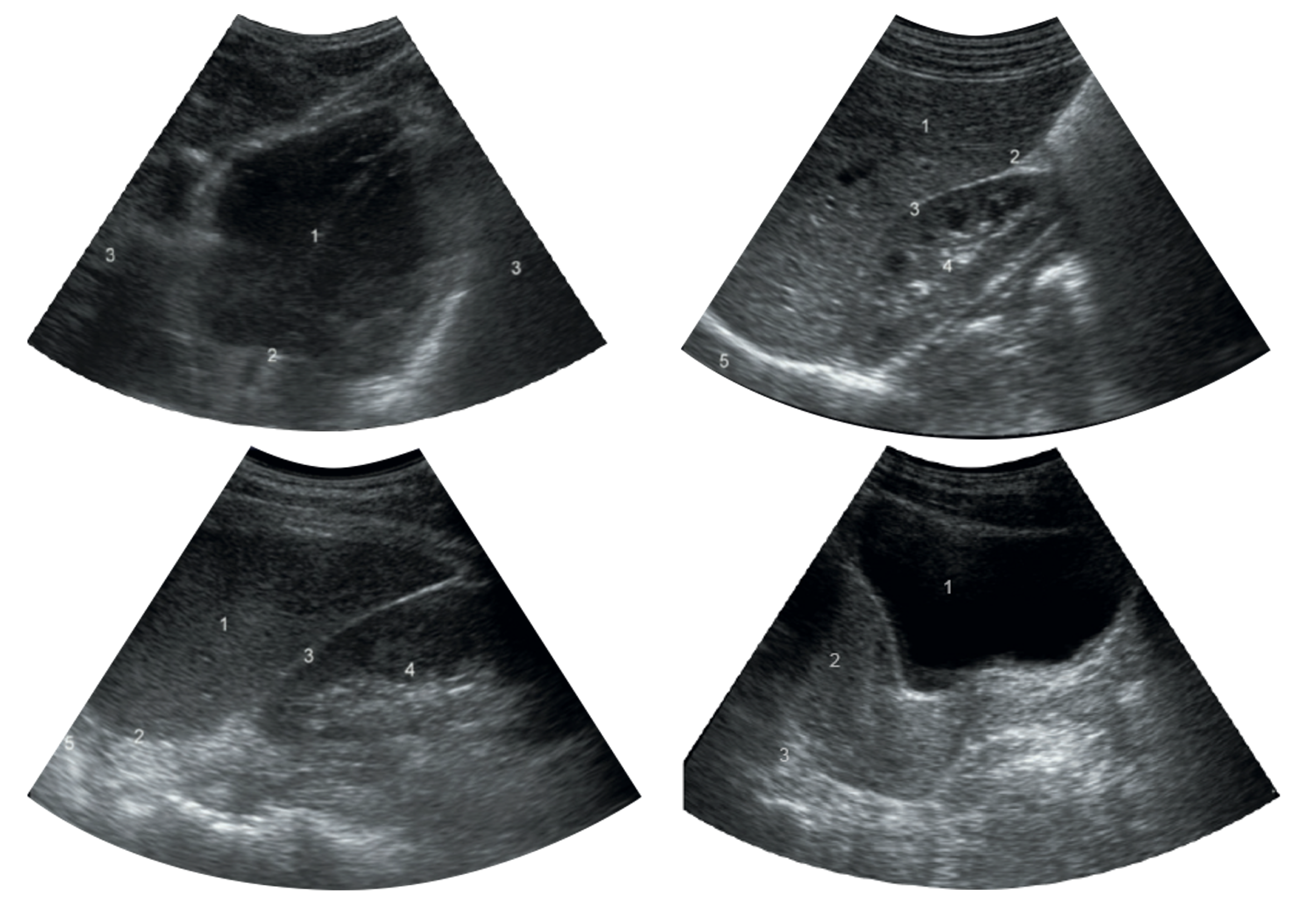

Supplement: Supplementary file 5 — Figure S5. Items for assessing static image interpretation. Learners had to label the correct sonoanatomical names for the numbers. [file MEDU-59-1105-s006.tif]
