# Supplementary material for: Should medical teachers spend more time modelling or coaching students? A dual eye‐tracking and randomised controlled study on peer instruction in sonography
Source: Med Educ. 2025 May 22;59(10):1105–16. doi: 10.1111/medu.15725 (PMC12438010; doi:10.1111/medu.15725)
Supplement: Supplementary file 6 — Table S1. Instructions for participants. [file MEDU-59-1105-s005.docx]

# Supplemental Data


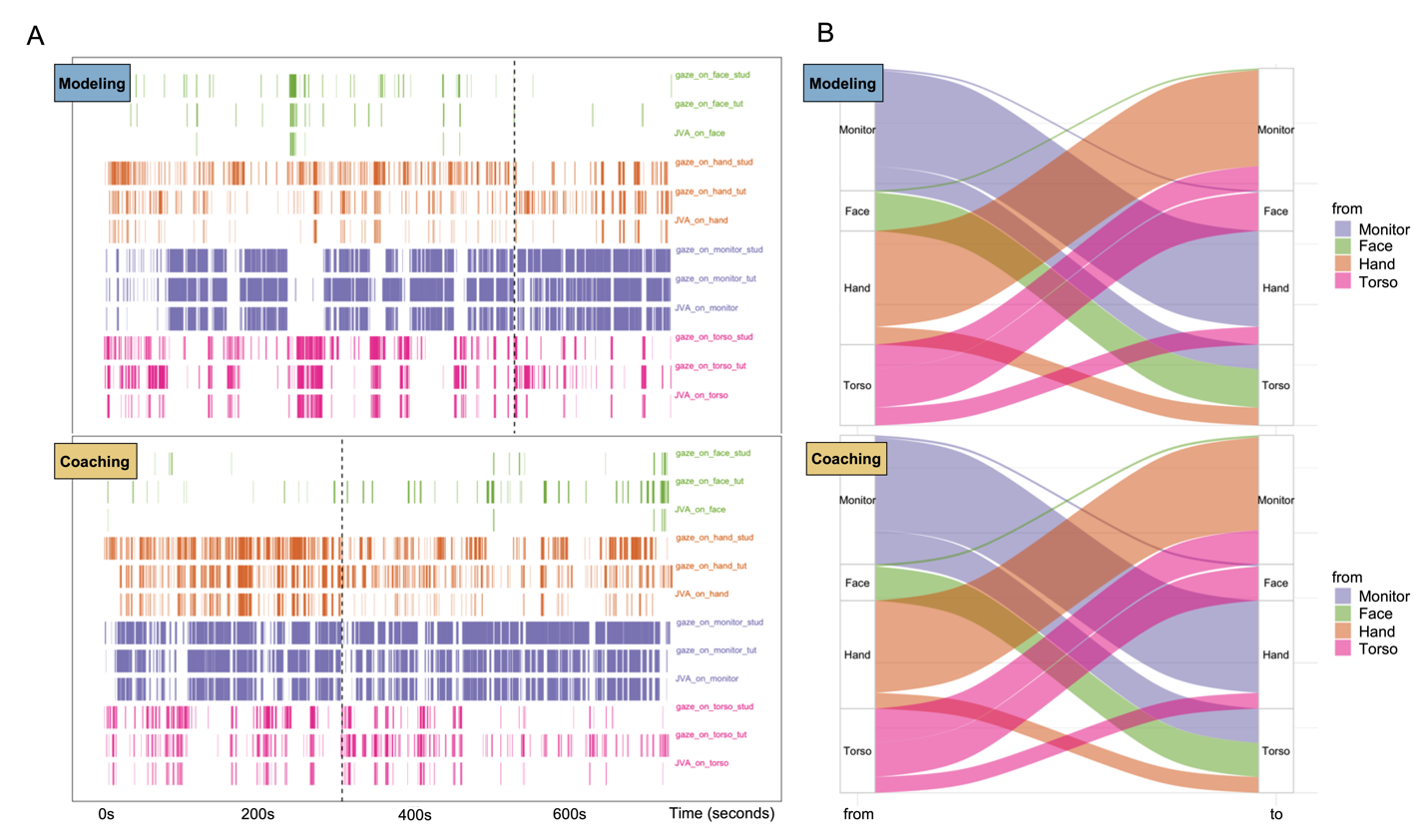


**Supplemental Figure 1.** (A) Panel showing eye movements of one dyad from each training Modeling (skyblue) and Coaching (copper). (B) Panel showing the transitions from one area of interest to another separately for both groups.


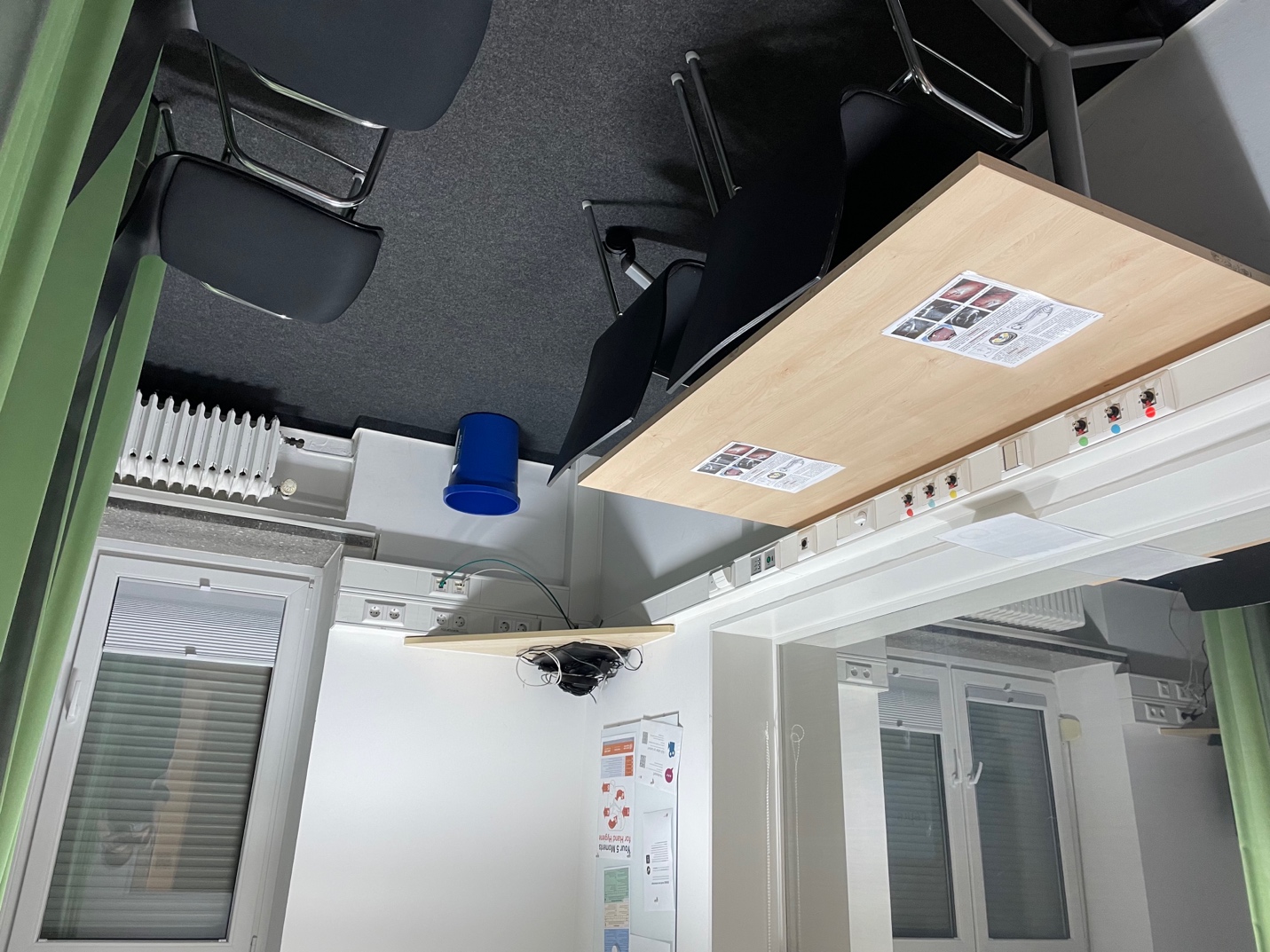


**Supplemental Figure 2.** Station 2 with a theoretical introduction on FAST sonography.


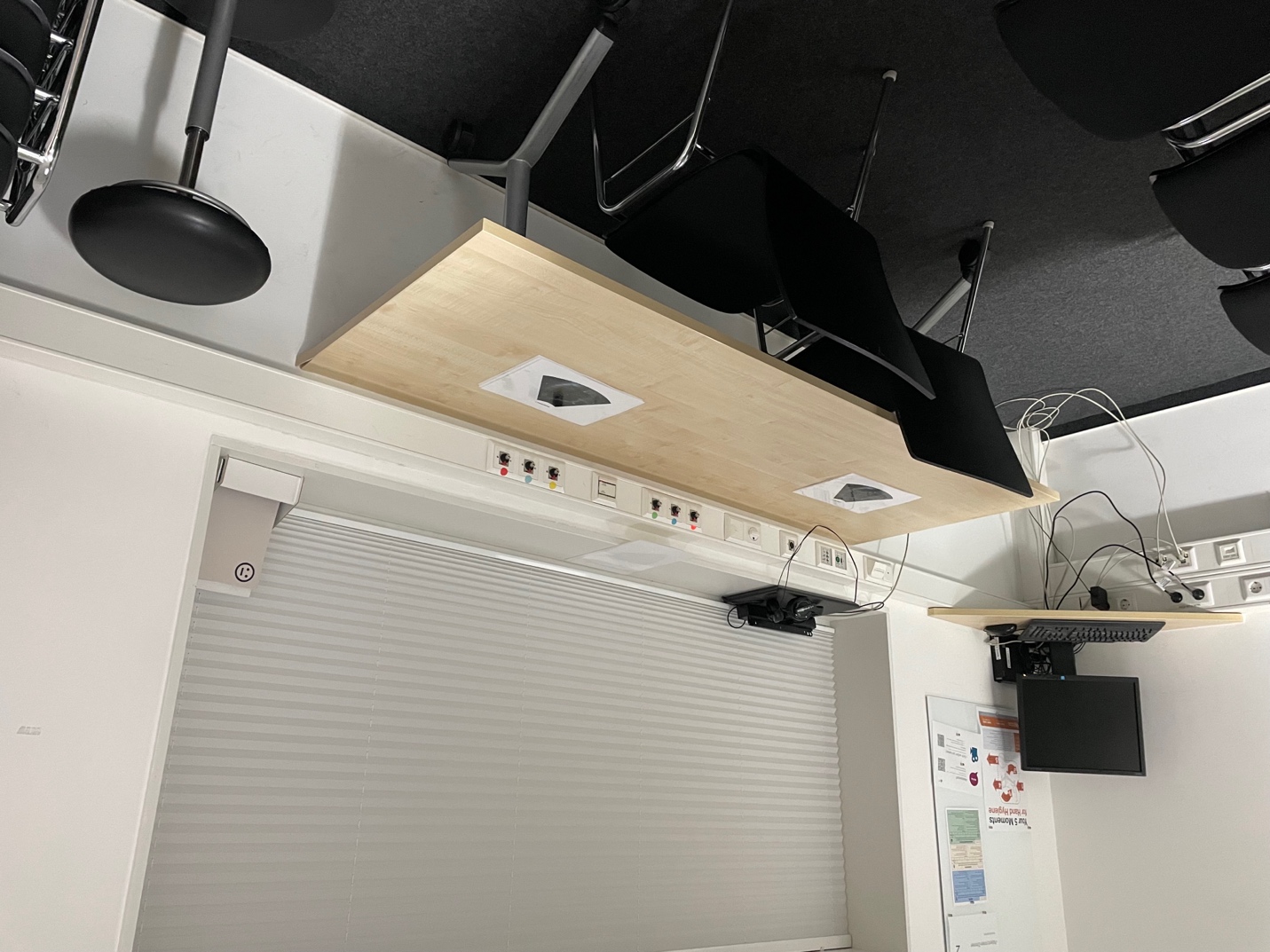


**Supplemental Figure 3.** Station 6 is shown, where learners interpreted sonographic images to assess their static image interpretation performance.


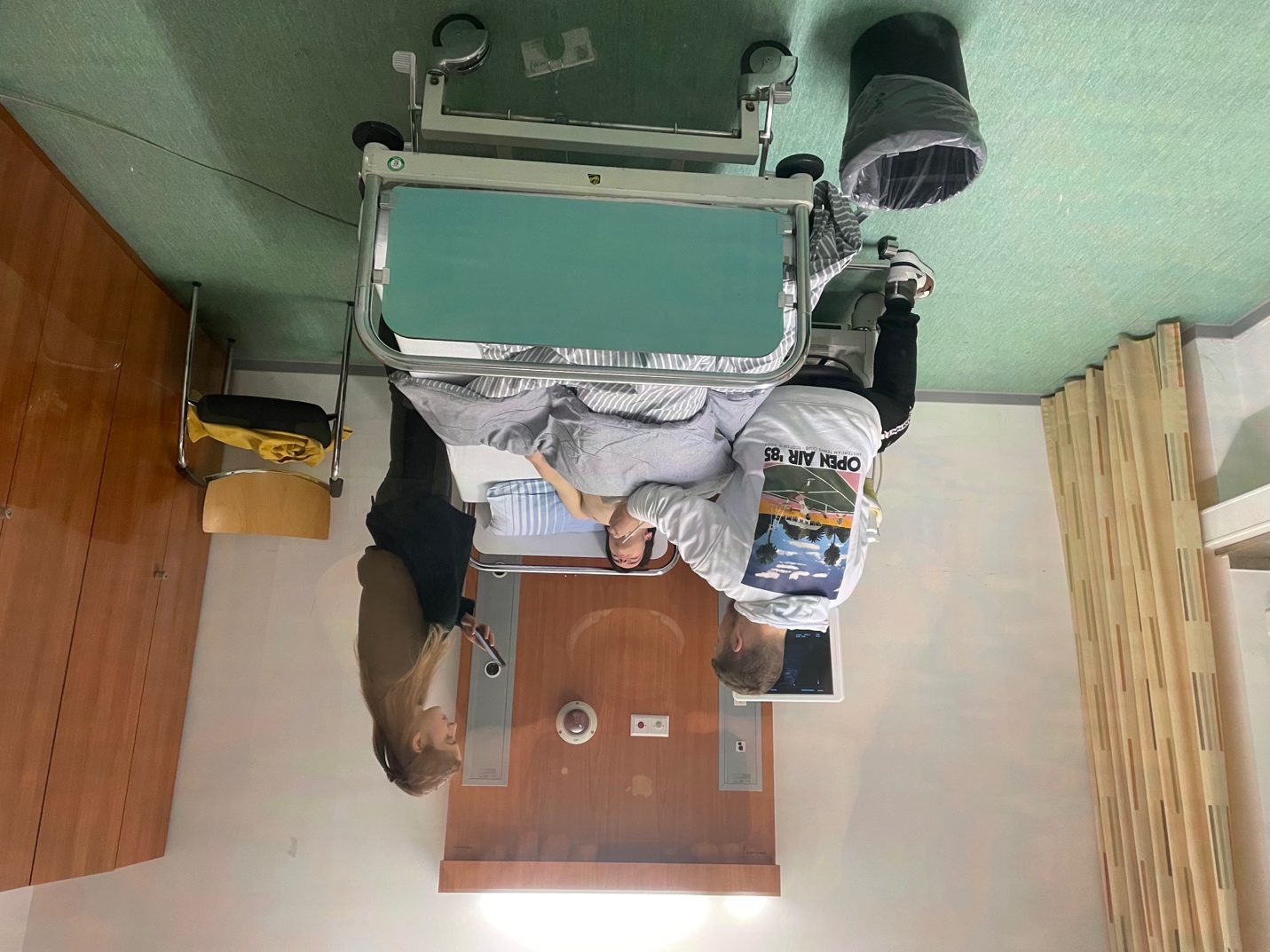


**Supplemental Figure 4.** Station 5 is shown, where the learner (left) performed an emergency sonography examination. The tutor (right), who was blinded to the intervention, assessed their practical performance and dynamic image interpretation performance based on a standardized checklist (OSCE).

| 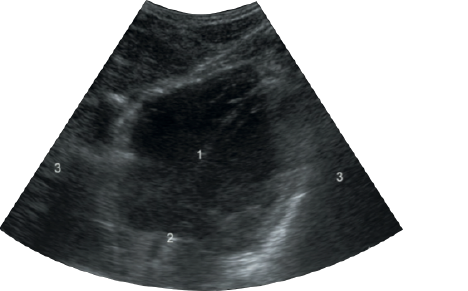 | 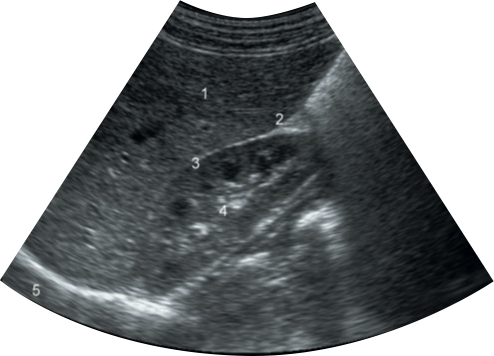 |
| --- | --- |
| 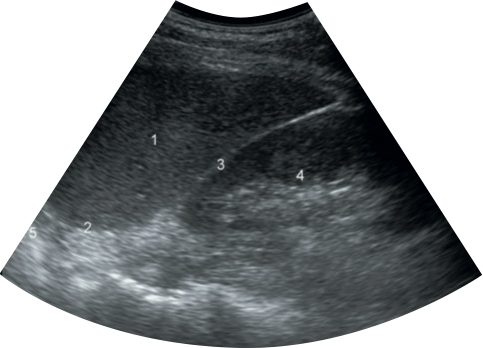 | 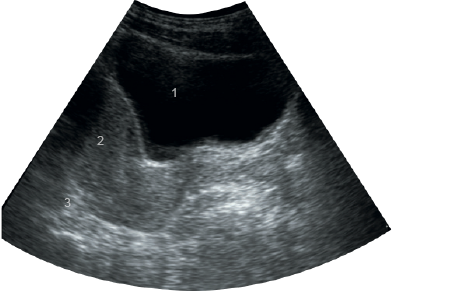 |
| **Supplemental Figure 5.** Items for assessing static image interpretation. Learners had to label the correct sonoanatomical names for the numbers. | |

| **Supplemental Table 1.**  Instructions for the participants | |
| --- | --- |
| *Begin* | “Thank you for participating in the study on practical learning environments in sonoanatomy. We offer you the opportunity to acquire or deepen basic sonoanatomical knowledge in an approximately 1-hour study. We monitor this process using mobile eye-tracking technology and questionnaires. The goal is to improve future learning environments. All collected data will be treated confidentially. There are no disadvantages to participating. Participation is voluntary. You can indicate at the start whether we may use your data for scientific purposes. For questions or concerns, contact XXX. If desired, we can send you the results after study evaluation.” |
| *Training Phase* | “In this section, you will receive a 12-minute ultrasound training with a clinical tutor. Your eyes will be tracked and the session will be video and audio recorded. Approach this training as you would in your student education environment, and feel free to discuss anything important to you. We know the glasses look a bit strange, but try to ignore them during the conversation. Try to keep your head steady so the glasses don't slip, though you can still gesture and nod naturally during conversation.Continue with the training until I return, in approximately 12 minutes.” |
| *Post-Training* | “Next, we would like to ask you some questions about the training.” |
